# Scientific Items

**Published:** 1877-04-20

**Authors:** 


### SCIENTIFIC ITEMS.

It is interesting to see how the same idea under specifically different forms is represented in the animal kingdom in the different portions of the earth. Take the idea which finds its expression in the ox, for example. In Africa we find the ox in the form of the Cape buffalo, very ferocious, and horns of great width. In India the Arni, whose enormous horns are ten feet apart from tip to tip; in Tartary, the grunting cow or yak, which has a long mane upon the back and a tail something like a horse, and in western North America, the Buffalo, etc.—Pop. Science Monthly.

THE TELEPHONE—An apparatus for transmitting by telegraph the sounds of the human voice, was invented by Prof. Bell of Boston, and has, by recent experiment, over a distance of 18 miles, in the presence of an audience in Boston, been successfully tested; so that the prophetic question of Job, (chap. xxxviii, 35.), propounded about 1500 years before the Christian era, may now be affirmatively answered: "Canst thou send lightnings, that they may go, and say unto thee, Here we are?"

A CHANGEABLE STAR.--M. Klein has discovered that the star Ursæ Majoris in "the Dipper," the one of the two pointers nearest the Pole Star, shows a periodical change of color, presenting a number of shades between intense fiery red and yellow, the length of the period being thirty-five days.

PLURAL BIRTHS.—In the last ten years plural births have increased in Prussia from 114 in 10,000 to 123. Of these 99 per cent. were twins. Triplets were somewhat less than 1 per cent. In over 6,000,000 births there were 79 cases of four at a birth, and one case of five.

